# Treatment with Peanut Sprout Root Extract Alleviates Inflammation in a Lipopolysaccharide-Stimulated Mouse Macrophage Cell Line by Inhibiting the MAPK Signaling Pathway

**DOI:** 10.3390/ijms20235907

**Published:** 2019-11-25

**Authors:** Yun Mi Lee, Eunjung Son, Dong-Seon Kim

**Affiliations:** Herbal Medicine Research Division, Korea Institute of Oriental Medicine, 1672 Yuseong-daero, Yuseong-gu, Daejeon 34054, Korea

**Keywords:** peanut sprouts root extract, inflammatory response, MAPK phosphorylation

## Abstract

Inflammation is a key response of the immune system to infection but aberrant inflammatory activity can lead to tissue damage and inflammatory diseases. Increasing evidence suggests that peanut sprout root extract (PSRE) has anti-inflammatory activity, and the aim of this study is therefore to investigate the effects of PSRE on the inflammatory response and the molecular mechanisms underpinning this effect in lipopolysaccharide (LPS)-stimulated RAW264.7 cells. Using a combination of cell viability, ELISA, and nitric oxide (NO) assays, together with Western blotting, we showed that PSRE effectively inhibited NO production in LPS-stimulated cells and significantly reduced the expression of pro-inflammatory cytokines, including IL-6, IL-1β, and PGE2, at a dose of 200 µg/mL of PSRE, whereas TNF-α expression tended to decrease under PSRE treatment. We also confirmed a dose-dependent and significant inhibition of iNOS and COX-2 protein expression. In addition, PSRE treatment induced anti-inflammatory effects by inhibiting the phosphorylation of MAPKs (ERK, JNK, and p38) and NF-κB activation. Our results indicate that the anti-inflammatory properties of PSRE may result from inhibition of the MAPK pathways, which are known promoters of cytokine secretion.

## 1. Introduction

Inflammation is a local response of the immune system to pathogens and damaged cells and is a vital defense mechanism [1]. However, an excessive or aberrant inflammatory response can be a major contributor to the pathophysiology of acute and chronic human diseases [2]. Macrophages play an important role in the inflammatory response and are naturally activated by exposure to bacterial lipopolysaccharide (LPS), which increases their capacity for phagocytosis during the removal of infectious agents. Activated macrophages overexpress several mediators of inflammation in response to LPS-induced cell injury, such as tumor necrosis factor-α (TNF-α), interleukin-1β (IL-1β), interleukin-6 (IL-6), prostaglandin E2 (PGE2), nitric oxide (NO), inducible nitric oxide synthase (iNOS), and cyclooxygenase (COX)-2 [3,4,5]. Members of the mitogen-activated protein kinase (MAPK) and nuclear factor-kappa B (NF-κB) signaling pathways have been shown to control the mechanisms of the inflammatory response in LPS-stimulated macrophages. The anti-inflammatory effects of suppressing cytokine production by inhibiting MAPK signaling and NF-κB transactivation have been considered targets for therapies aimed at reducing LPS-induced inflammation, and the levels of these cytokines have been frequently used as markers to assess the activity of anti-inflammatory drugs.

Recently, there has been increasing interest in the health benefits of peanut sprouts (PS), which are germinated peanut seeds. Sprouted peanuts have a lower amount of fats and contain higher amounts of carbohydrates, amino acids, and minerals compared to the seed. They also contain a wide variety of phenolic compounds, including a high content of resveratrol, protocatechuic acid, gallic acid, and caffeic acid [6]. Previous studies using PS extract have reported its regulatory effects in the context of cisplatin-induced ototoxicity, obesity, and the inflammatory response in mouse skin and compound 48/80-treated HaCaT keratinocytes [7,8,9]. However, the activity of LPS-stimulated macrophages anti-inflammatory effects generated by PS extract still remain unknown. We therefore examined the anti-inflammatory effects of PS root extract (PSRE) in LPS-stimulated RAW264.7 macrophage cells.

## 2. Results

### 2.1. Chemical Profile of PSRE

**Figure 1:**
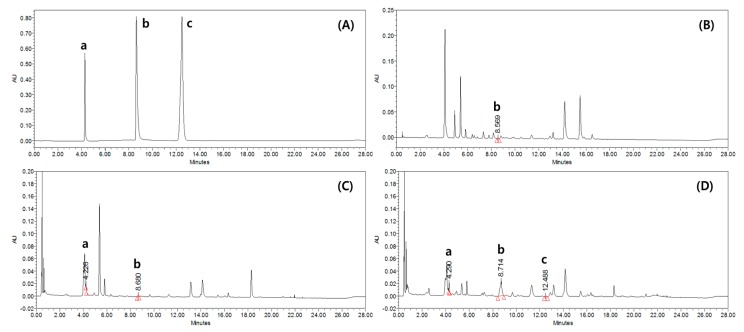
Representative Ultra Performance Liquid Chromatography (UPLC) chromatogram at 320 nm: (**A**) standard solutions of caffeic acid (a), cichoric acid (b), and resveratrol (c), (**B**) day 0 peanut sprout root extract (PSRE), (**C**) day 3 PSRE, and (**D**) day 9 PSRE.

Several published studies using PS extracts have analyzed their chemical composition and described a wide range of compounds, the levels of which vary over the course of germination. We therefore began our investigations by examining the chemical profile of the PSRE generated in this study. Based on the absorption profile and retention time, cichoric acid was identified as a marker compound of peanut sprout roots germinated for 9 days, with day 9 PSRE containing 0.998 ± 0.011 mg/g of cichoric acid (8.7 min). Day 9 PSRE also contained 0.108 ± 0.009 mg/g of caffeic acid and 0.011 ± 0.000 mg/g of resveratrol (Figure 1).

### 2.2. Effect of PSRE on Viability, NO Production and NF-κB Activation of RAW264.7 Macrophages

**Figure 2:**
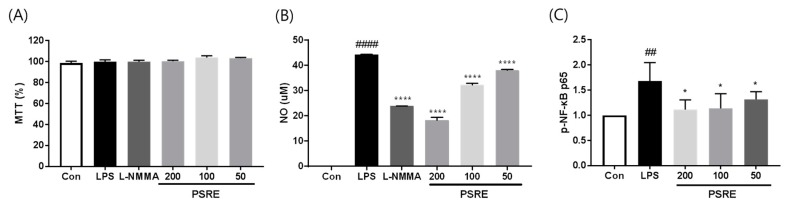
Effect of PSRE on viability, NO production, and NF-κB activation of LPS-induced RAW264.7 macrophages. Cells were pretreated with PSRE (0, 50, 100, or 200 μg/mL) for 2 h and then treated with LPS (0.5 μg/mL) for 22 h. (**A**) Cell viability was measured using an MTT (3-(4,5-dimethylthiazol-2-yl)-2,5-diphenyltetrazolium bromide) assay. (**B**) NO production was measured using a Griess reagent assay. L-NG-monomethyl arginine (L-NMMA) was used as a positive control. (**C**) NF-κB activation was measured using an ELISA. Values are expressed as means ± SD (*n* = 3). ^##^ *p* < 0.01, ^####^ *p* < 0.0001 vs. vehicle control cells. * *p* < 0.05, **** *p* < 0.0001 vs. LPS-treated cells.

To determine the effect of PSRE treatment on cell viability, NO production and NF-κB activation during inflammation, RAW264.7 cells were pre-treated with a range of PSRE concentrations, followed by treatment with LPS to induce an inflammatory response. MTT assays showed that treatment with PSRE alone for 24 h at the maximum concentration of 200 μg/mL produced no significant change in cell viability compared to that in an untreated control group. Therefore, we used concentrations of 200 μg/mL or less for all subsequent experiments (Figure 2A).

NO is a signaling molecule which plays an important role in the inflammatory response. To examine whether PSRE treatment could modulate NO production, we measured the NO secretion in LPS-induced RAW 264.7 cells after PSRE treatment, using a Griess reagent assay. As shown in Figure 2B, LPS treatment significantly induced NO production compared to that in the untreated control, while cells pretreated with PSRE demonstrated a significant inhibition of NO production in a dose-dependent manner.

Since NF-κB was identified as an important transcription factor that controls several pro-inflammatory mediators, we investigated the activation of NF-κB by ELISA and the results are shown in Figure 2C. PSRE reduced NF-κB levels in a dose dependent manner in LPS-induced RAW 264.7 cells.

### 2.3. Effect of PSRE on the Expression of Inflammatory Cytokines in RAW264.7 Macrophages

**Figure 3:**
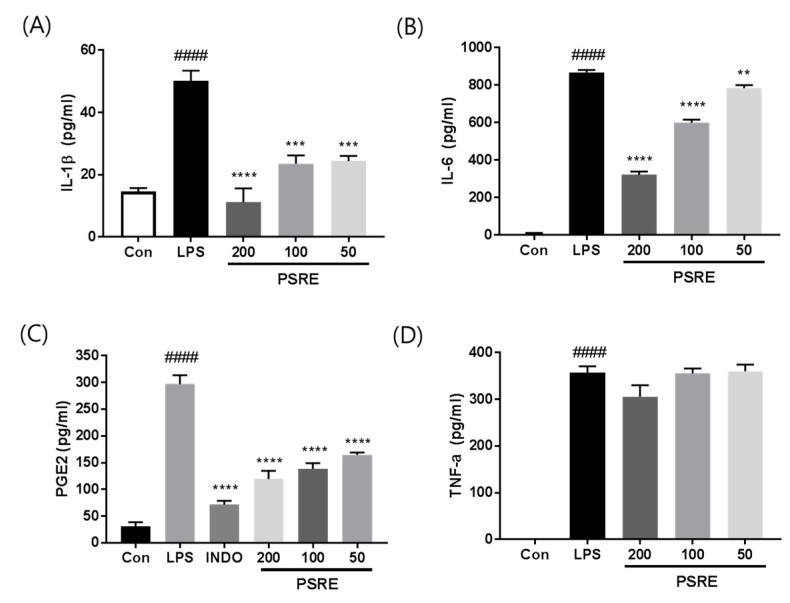
Effect of PSRE on IL-1β, IL-6, PGE2, and TNF-α production in LPS-stimulated RAW264.7 macrophages. Cells were pretreated with PSRE (0, 50, 100, or 200 μg/mL) for 2 h and then with LPS (0.5 μg/mL) for 22 h. The supernatants were collected and subjected to ELISA for (**A**) IL-1 β, (**B**) IL-6, (**C**) PGE2, and (**D**) TNF-α. Indomethacin (INDO), a potent inhibitor of PGE2 synthesis in vitro, was used as a positive control. The values are expressed as the mean ± SD (*n* = 3). ^####^ *p* < 0.0001 vs. vehicle control cells. ** *p* < 0.01; *** *p* < 0.001; **** *p* < 0.0001 vs. LPS-treated cells.

To determine whether the ability of PSRE to inhibit inflammatory signaling corresponded to a reduction in the secretion of pro-inflammatory cytokines, we investigated cytokine secretion in LPS-activated macrophages using ELISA. As shown in Figure 3, at a dose of 200 µg/mL, PSRE treatment dramatically decreased the expression of the pro-inflammatory cytokines IL-1β, IL-6, and PGE2 by 77.7%, 63%, and 60%, respectively. TNF-α levels were markedly increased in LPS-treated control cells but pre-treatment with PSRE tended to mitigate this upregulation.

### 2.4. Effect of PSRE on COX-2 and iNOS Protein Expression

**Figure 4:**
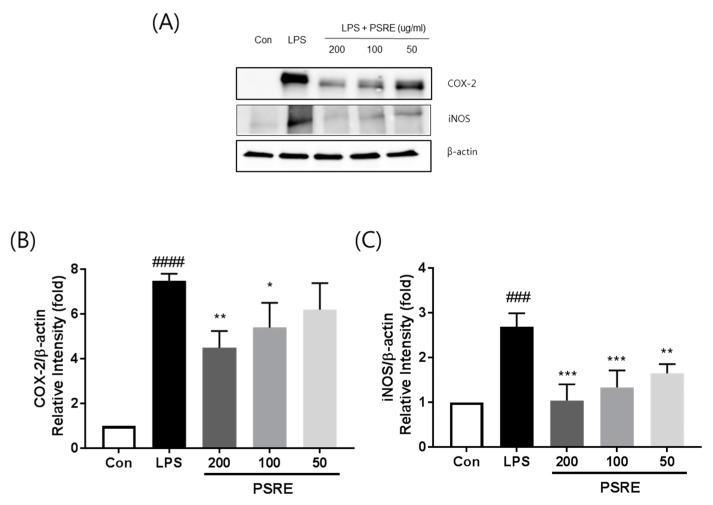
Effect of PSRE on COX-2 and iNOS expression in RAW 264.7 cells. (**A**) Total protein was extracted and subjected to Western blot analysis. Relative amount of each protein was determined by densitometric analysis. The levels of (**B**) COX-2 and (**C**) iNOS were estimated according to the value of each control. The values are expressed as the mean ± SD (*n* = 3). ^###^ *p* < 0.001, ^####^ *p* < 0.0001 vs. vehicle control cells. * *p* < 0.05, ** *p* < 0.01, *** *p* < 0.001vs. LPS-treated cells.

Two other common mediators of inflammation are COX-2 and iNOS. To evaluate whether PSRE influences COX-2 and iNOS expression, we performed Western blot analysis. LPS-stimulated cells exhibited a significant increase in COX-2 and iNOS expression, when compared to the untreated control. Treatment with PSRE greatly down-regulated the production of COX-2 and iNOS stimulated by LPS in a concentration-dependent manner, as shown in Figure 4.

### 2.5. Effect of PSRE on MAPK Phosphorylation

**Figure 5:**
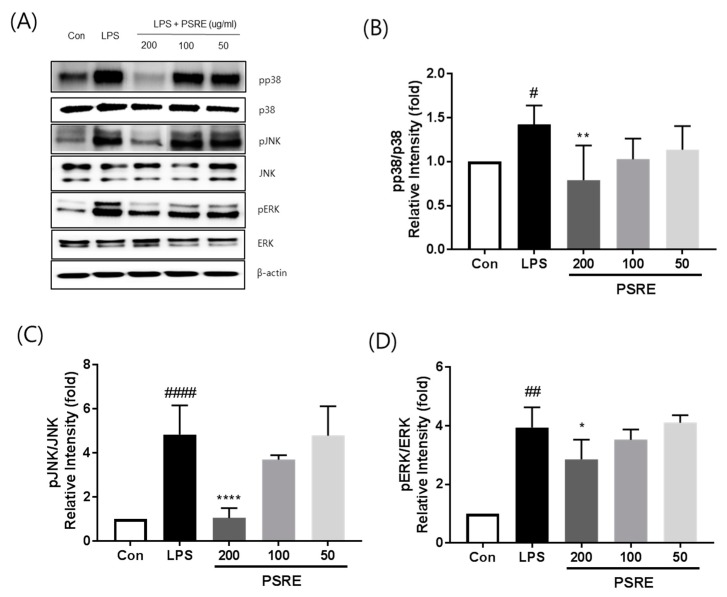
Effect of PSRE on MAPK phosphorylation in RAW 264.7 cells. (**A**) Total protein was extracted and subjected to Western blot analysis. Relative amount of each protein was determined by densitometric analysis. The levels of (**B**) p38, (**C**) JNK, and (**D**) ERK were estimated according to the value of each control. The values are expressed as the mean ± SD (*n* = 3). ^#^ *p* < 0.05, ^##^ *p* < 0.01, ^####^ *p* < 0.0001 vs. vehicle control cells. * *p* < 0.05, ** *p* < 0.01, **** *p* < 0.0001 vs. LPS-treated cells.

While a number of signaling pathways have been shown to mediate inflammation, one of the most well-known is the MAPK signaling pathway. We therefore used Western blot analysis to determine whether PSRE treatment of activated macrophages affected the phosphorylation of the upstream MAPK kinases, namely p38 MAPK, ERK, and JNK. As shown in Figure 5, LPS treatment elevated the phosphorylation of p38 MAPK, ERK, and JNK. In addition, the phosphorylation of p38 MAPK and ERK was remarkably attenuated by PSRE treatment. These results suggest that PSRE treatment blocks the p38 MAPK, ERK, and JNK pathways to exert its anti-inflammatory effects on LPS-treated RAW 264.7 cells.

## 3. Discussion

Inflammation plays a pivotal role in the defense response of many organs to pathogenic stimuli, such as bacterial infection [10]. LPS is the immunologically active component of Gram-negative bacterial cell walls and is thus commonly employed as a proxy for infection. LPS stimulates macrophages to induce numerous inflammatory mediators including NO, IL-1β, TNF-α, PGE2, and cyclooxygenase [11,12,13,14]. NO is a vital cellular signaling molecule involved in inflammation through iNOS upregulation, and the over-production of NO has been linked to various inflammatory disorders [15]. In addition, NO induces PGE2 production, resulting in accelerated inflammatory responses through its activation of COX-2 [16].

Pro-inflammatory cytokines such as TNF-α, IL-1 β, and IL-6 act as messengers to stimulate the inflammatory process [17]. TNF-α induces synergy in NO production in LPS-stimulated macrophages, causing inflammatory responses such as vasodilatation, edema, and fever [18]. IL-1β is activated by caspase-1, and is critical for the response to infection as it influences the production of NO [19]. In addition, IL-6 is a pro-inflammatory cytokine with many functions, including propagating chronic inflammation and inducing acute inflammation [20]. Therefore, targeting the inflammatory mediators and cytokines is a useful strategy in anti-inflammatory therapy [21]. In the present study, we provided evidence that suppression of NO and PGE2 production by PSRE treatment correlated with a decrease in the expression levels of COX-2 and iNOS. In addition, we also found that PSRE inhibits TNF-α, IL-1β, and IL-6 production in LPS-stimulated RAW 264.7 cells.

MAPK signaling pathways play an important role in the up-regulation of COX-2, iNOS, and inflammatory cytokines induced by various stimuli [22,23,24]. Thus, inactivation of MAPKs might inhibit the synthesis of pro-inflammatory cytokines and their signaling. Therefore, we investigated whether the phosphorylation of JNK, ERK, and p38 MAPK was inhibited in LPS-stimulated RAW264.7 cells. Our data showed that PSRE had remarkable inhibitory effects on LPS-stimulated MAPK phosphorylation at concentrations of 200 μg/mL.

The MAPK signaling pathways regulate transcription factors, such as NF-κB, that transcriptionally activate many genes involved in the regulation of immune responses, cell adhesion, and survival [25]. During the inflammatory process, NF-κB is translocated to the nucleus and transcriptionally activates various inflammatory cytokines such as iNOS, COX-2, TNF-α, and IL-1β [26,27]. Our data show that PSRE had a significant inhibitory effect on the LPS-stimulated activation of NF-κB. These results suggest that PSRE plays an anti-inflammatory role in LPS-induced macrophage cells. Indeed, our promising in vitro data could form the basis of future studies examining the effect of PSRE in animal models of inflammation-related disease [9].

Research on the health benefits of PS has been gaining increasing prominence, revealing that PS extracts have considerable antioxidant potential. The sprouting process promotes significant changes in bioactive compounds that contain various types of phenolic compounds, many of which are antioxidants [28]. Limmongkon et al. reported that the peanut seed coat, germinated kernel, and germinated root were abundant in a variety of phenolic substances, such as caffeic acid, coumaric acid, hesperidin, transarachidin-1, arahypin 2, arahypin 3, resveratrol, 4-isopentadienyl-3,5,3′,4′-tetrahydroxystilbene (IPP), trans-3′-isopentadienyl-3,5,4′-trihydroxystilbene (IPD), transarachidin-1, trans-arachidin-2, and trans-arachidin-3 [29].

In our study, cichoric acid was barely detectable in PSRE on day 0 and day 3, but was detectable at a level of 0.998 ± 0.011 mg/g on day 9 of germination. As a result, we speculate that the appearance of cichoric acid may be due to increased levels of a compound that is converted during the germination process. In addition, our analyses revealed the presence of 0.108 ± 0.009 mg/g of caffeic acid and 0.011 ± 0.000 mg/g of resveratrol in day 9 PSRE. To our knowledge, this is the first paper to confirm the presence of cichoric acid in PSRE. Cichoric acid is a natural phenolic compound obtained in a number of plants, such as chicory (*Cichorium intybus*) and Echinacea (*Echinacea purpurea*) and it possesses antioxidant, anti-inflammatory, antiviral, and analgesic activities in vitro and in vivo [30,31,32]. However, cichoric acid has not been reported to have effects against inflammation in LPS-induced macrophages. Further investigation into the effects of this compound on inflammation in LPS-induced macrophages is therefore required.

## 4. Materials and Methods

### 4.1. PSRE Preparation

Peanut (*Arachis hypogaea* L.) seeds were washed, incubated in water at 20 °C for 18 h, and then germinated in a hydroponics system at 80% humidity at 26 °C in the dark. After harvesting 3 to 9 days after germination, peanut sprout roots were dried, finely powdered, and extracted with 70% ethanol for 3 h at reflux, and these extracts were concentrated under reduced pressure, freeze dried, and stored at 4 °C.

### 4.2. Chemical Profiling of PSRE

A Waters ACQUITY Ultra Performance Liquid Chromatography (UPLC) system equipped with a quaternary pump, auto-sampler, and photodiode array detector with an ACQUITY UPLC^®^ BEH C18, 100 × 2.1 mm, 1.7 μm column was used for the analysis (Waters, MA, USA). An elution with solvent A (0.1% phosphoric acid) and solvent B (Acetonitrile) in a gradient elution at a flow rate of 0.5 mL/min was carried out as follows: 0–2 min, 5–5% B; 2–4 min, 5–8% B; 4–20 min, 8–26% B; 20–24 min, 26–100% B; 24–26 min, 100–5% B; 26-min, 5–5% B. The detection wavelength was set at 320 nm. The column temperature was kept at 40 °C and the injection volume was 2 µL.

### 4.3. Cell Culture

The murine macrophage RAW264.7 cell line, purchased from American Type Culture Collection, was cultured in Dulbecco’s Modified Eagle’s medium (DMEM), supplemented with 10% heat inactivated fetal bovine serum (FBS), 1% penicillin (100 IU/mL) at 5% CO_2_ and 37 °C. The medium was replaced with serum-free DMEM medium. PSRE (50, 100, and 200 µg/mL) was dissolved in 100% DMSO, and each concentration was diluted to a final DMSO content of 0.1% when used to treat cells for 2 h. The control groups were treated with vehicle solution. LPS (0.5 µg/mL Sigma-Aldrich Chemical Co., St. Louis, MO, USA) was added in the presence or absence of PSRE for an additional 22 h to stimulate the cells.

### 4.4. Cell Viability

Cell viability assays were performed to determine the cytotoxicity of PSRE using 3-(4,5-dimethylthiazol-2-yl)-2,5-diphenyltetrazolium bromide (MTT) (Sigma-Aldrich Chemical Co., St. Louis, MO, USA). RAW 264.7 cells were incubated with various concentrations of PSRE (0–200 µg/mL) for 24 h and the treatment medium then completely replaced with MTT solution. MTT was dissolved in serum-free DMEM at concentration of 0.5 mg/mL, and 100 μL of this solution was added to cell cultures for 4 h in 96-well culture plates. The plates were removed from the incubator and the formazan crystals were dissolved by the addition of 100 μL of dimethyl sulfoxide. The absorbance at 570 nm was read on a microplate reader (Bio-Rad, Hercules, CA, USA) as a measure of cell viability. The absorbance was normalized to that of cells incubated in the control medium, and these cells were considered 100% viable. The percentage of cell viability was calculated as follows: (Mean optical density (OD) in PSRE-treated cells/Mean OD in untreated cells × 100).

### 4.5. NO and Inflammatory Cytokine Assays

NO production was analyzed using the Griess Reagent System, according to the manufacturer’s instructions (Promega, Madison, WI, USA). Briefly, the accumulation of nitrite in culture supernatants was detectable via a chemical reaction with the Griess reagents which produces a dye measurable by spectrophotometry. L-NG-monomethyl arginine (L-NMMA) was used as a positive control. Collected cell supernatants were reacted with Griess reagent, and the absorbance was measured at 540 nm. For the measurement of NF-κB levels, cells were lysed and processed according to the manufacturer’s instructions using the Pathscan phospho-p65 (Ser536) ELISA kit (Cell Signaling). The levels of IL-1β, IL-6, TNF-α, and PGE2 were measured using ELISA kits from R&D Systems (Minneapolis, MN, USA) according to the manufacturer’s protocol. Indomethacin (INDO), a potent inhibitor of PGE2 synthesis in vitro, was used as a positive control.

### 4.6. Western Blot Analysis

Cell lysates were prepared from RAW264.7 cells in 1× Laemmli lysis buffer and boiled for 10 min. The samples (20 g) were diluted with 1× lysis buffer, separated by electrophoresis (4.5% to 15% gradient), and transferred onto polyvinylidene fluoride (PVDF) membranes. The membranes were reacted with primary antibodies against COX-2, iNOS, p-p38, p38, pJNK, JNK, pERK, ERK, and β-actin (1:1000 dilution) (Cell Signaling Technology, Beverly, MA, USA). Specifically, bound horseradish peroxide-conjugated secondary antibodies were detected using an enhanced chemiluminescence detection system (Amersham Bioscience, Buckinghamshire, UK). Protein expression levels were determined by the analysis of the signals captured on the PVDF membrane using an image analyzer (LAS-3000, Fujifilm, Tokyo, Japan).

### 4.7. Statistical Analysis

The results are expressed as the mean ± standard deviation (SD) and analyzed using one-way analysis of variance (ANOVA) followed by Dunnett’s tests for multiple comparisons or unpaired Student’s *t*-tests for two-group comparisons. All analyses were performed using Prism 7.0 (GraphPad Software, San Diego, CA, USA), and *p*-values < 0.05 were considered statistically significant.

## 5. Conclusions

The results of our study show that PSRE treatment suppressed the inflammatory mediator (NO and PGE2) production, likely through decreased expression of iNOS and COX-2, and inhibited inflammatory cytokines such as TNF-α, IL-1β, and IL-6 through the inhibition of MAPK signaling and NF-κB activation in LPS-induced macrophages. These findings suggest that PSRE may be a potential therapeutic agent for the treatment of inflammation-related diseases.

## Abbreviations

PSRE	Peanut Sprouts Root Extract
LPS	Lipopolysaccharide
NO	Nitric oxide
TNF-α	Tumor necrosis factor-α
IL-1β	Interleukin-1β
IL-6	Interleukin-6
PGE2	Prostaglandin E2
iNOS	Inducible nitric oxide synthase
COX-2	Cyclooxygenase 2
MAPK	Mitogen-activated protein kinase
ERK	Extracellular signal-regulated kinase
JNK	c-Jun n-terminal kinase
